# Health Biomarkers in Adults Are More Closely Linked to Diet Quality Attributes Than to Plant-Based Diet Categorization

**DOI:** 10.3390/nu11061427

**Published:** 2019-06-25

**Authors:** Selicia Mayra, Noel Ugarte, Carol S. Johnston

**Affiliations:** College of Health Solutions, Arizona State University, Phoenix, AZ 85004, USA; smayra@asu.edu (S.M.); noelu104@gmail.com (N.U.)

**Keywords:** diet quality, vegetarian diets, plant-based diet, health biomarkers, chronic disease risk, REAP-S

## Abstract

Although plant-based diets are promoted for healthy outcomes, these diets are not synonymous with high-quality diets. Plant-based diets can include highly processed, less healthful foods, including savory snacks, pastries, and sugary fruit drinks. This cross-sectional study examined the diet quality of vegetarian and omnivorous adults, matched for gender, age, and adiposity, and related diet quality to standard health biomarkers. Diet quality was assessed using the Rapid Eating and Activity Assessment for Participants Short Version questionnaire. Participants (17 vegetarians and 16 omnivores; 28.2 ± 8.9 years; 22.5 ± 2.7 kg/m^2^) were non-smokers and healthy by self-report. The median duration of adherence to the vegetarian diet was 27 months. Physical activity level and diet quality did not differ significantly between diet groups. Moreover, health biomarkers did not differ by diet groups. When participants were regrouped by low versus high diet quality, health biomarkers differed significantly between groups for fasting insulin, HOMA-IR, triglyceride (TG)/HDL ratio, and blood folate, with more favorable levels in the group with high diet quality. These data suggest that health biomarkers more closely align with diet quality attributes than with plant-based diet categorization. Thus, messaging focused on healthy diet attributes may lead to better health outcomes than the simple promotion of plant-based diets.

## 1. Introduction

Plant-based diets rich in fruits, vegetables, whole grains, nuts, and legumes are consistently linked to reduced risk for chronic disease, improved cognition, and longevity [1,2,3,4,5,6,7]. A vegetarian eating pattern with little to no flesh foods is typically considered the model plant-based diet, and experts suggest that adopting a vegetarian-like eating pattern is likely the most efficacious diet choice [8,9]. However, although plant-based diets are promoted for healthy outcomes, these diets are not synonymous with a high-quality eating pattern. In fact, plant-based diets can include less healthful plant foods including savory snacks, desserts, and sugar-sweetened beverages as well as highly processed convenience foods. To more fully investigate the link between plant-based diets and disease risk, recent investigations have categorized plant-based diets as ‘healthful’ (plant-based diets rich in whole grains, fruits, vegetables, nuts, legumes, vegetable oils, tea/coffee) or ‘unhealthful’ (plant-based diets rich in fruit juices, sugar-sweetened beverages, refined grains, potatoes, sweets/desserts) [10,11]. For these investigations, the plant-based diet categories were applied to three large ongoing prospective cohorts (the Nurses’ Health Study 1 and 2 and the Health Professionals Follow-up Study) encompassing nearly 290,000 enrollees with 8631 and 16,162 incident cases of cardiovascular disease and type 2 diabetes, respectively. In both analyses, only the healthful plant food diets were associated with significant reductions in the hazard ratios for disease incidence (–25% to –34%). Conversely, the unhealthful plant food diets were directly related to the risk for the incidence of cardiovascular disease (+32%) and type 2 diabetes (+16%).

Diet quality assessment is another strategy for identifying eating patterns linked to favorable health outcomes [12,13]. The Healthy Eating Index (HEI, debuted in 1989) is among the most used indices to evaluate diet quality in clinical research [14]. Since this index closely aligns with the U.S. Dietary Guidelines, it has been modified over the years to align with revisions of the Dietary Guidelines (retitled: HEI-2005, HEI-2010, and HEI-2015). In terms of administering and scoring, the Rapid Eating and Activity Assessment for Patients (REAP) [15] is a simpler measure than the HEI. Moreover, as indicated by several validation trials, both the HEI and REAP are comparable in terms of diet quality assessment [16,17]. Importantly, unlike the HEI-2010, REAP scores strongly correlate with other indicators of diet quality including the nutrient density of the diet, dietary potential renal acid load, urinary pH, and plasma vitamin C [17].

This cross-sectional study examined the diet quality and blood metabolites of vegetarian and omnivore adults (matched by gender, age, and adiposity) to determine how adherence to plant-based diets compared to high-quality eating patterns.

## 2. Materials and Methods

### 2.1. Participants

Non-smoking, omnivorous or vegetarian adults (18 to 65 years; diet adherence ≥6 months by self-report), from a campus population and interested individuals from the surrounding community in Phoenix, Arizona, were recruited using online media to complete a short online diet and demographics survey. A vegetarian was defined as following a lacto and/or ovo vegetarian or vegan diet for at least six months with total exclusion of flesh foods (including fish) for at least six months. An omnivore was defined as following a diet that included flesh foods daily, for at least six months. Each vegetarian recruit was matched by gender, age, and adiposity to an omnivore recruit. These pairs were invited to meet with study investigators and enroll in the study. Pregnant, recently pregnant (past three months), or lactating women, those with acute or chronic health conditions, and individuals exercising >30 min daily were excluded from the study. The study was approved by the University Institutional Review Board, and all participants provided written informed consent.

### 2.2. Study Design

For this cross-sectional, differential research trial, participants completed questionnaires covering demographics and a short health history. Height was recorded using a wall-mounted stadiometer, body weight was measured using a calibrated scale (model TBF-300A, Tanita Corporation, Tokyo, Japan), and body mass index (BMI; kg/m^2^) was computed. Waist measurements were taken using a flexible tension tape at the minimal circumference. Physical activity was recorded in metabolic equivalent of task (METS) [18], and diet quality was assessed using the Rapid Eating and Activity Assessment for Participants Short Version (REAP-S) questionnaire, which was slightly modified to include vegetarian food items. If the participant had fasted (no food or drink, except for water, for ≥10 h), a venous blood sample was collected from the antecubital vein. If necessary, the participant returned within several days to provide a fasting blood sample. Blood samples were quickly processed, and plasma was stored at −80 °C until analysis for total cholesterol, LDL and HDL cholesterol, triglycerides (TG), fasting blood glucose, and insulin. Plasma lipids and serum glucose were measured using a point-of-care COBAS C111 chemistry random access autoanalyzer (Roche Diagnostics, Indianapolis, IN, USA). Insulin concentration was measured by radioimmunoassay (Millipore, St. Charles, MO, USA). HOMA-IR was calculated as fasting insulin (mU/mL × fasting glucose mg/dL). A standard radioimmune assay system was used to measure plasma total folate (SimulTRAC-SNB Vitamin B12/Folate RIA Kit, item number 06B264806), a surrogate marker of diet quality [19].

### 2.3. Dietary Index

A modified version of the shortened REAP questionnaire (REAP-S) was completed for the previous week’s intake and was scored by summing responses to 15 questions (the 13 original questions plus 2 additional questions) [16]. The first few questions of the survey pertained to breakfast consumption as well as dining-out and ordering-in patterns. The remaining questions pertained to the consumption of whole grain products, fruits, vegetables, dairy products, meats, poultry or fish, processed meats, fried foods including chicken and French fries, snack items including potato chips and crackers, spreads including butter and margarine, sweets including cakes and cookies, and sugar-sweetened beverages including non-diet soda, and fruit drinks. The two questions added to the original survey queried about meals prepared from scratch and the use of frozen dinners. For vegetarian participants, one question on dairy-free alternatives and one question on meat analogs (e.g., tofu, seitan, and tempeh) replaced the questions on dairy and meat products that were completed by omnivore participants. For lacto vegetarians, however, the question on dairy consumption was retained. Responses of ‘usually/often’ received 1 point, ‘sometimes’ received 2 points, and ‘rarely/never’ or ‘does not apply to me’ received 3 points. Possible scores ranged from 15–45, and higher scores were indicative of better diet quality.

### 2.4. Statistical Analyses

Data are reported as mean ± SD; non-normal data were log-transformed prior to analysis. A significant multivariate analysis of variance test preceded univariate analyses for the anthropometric analyses and the blood marker analyses. A 2-way analysis of variance test was used to examine the interaction between diet group and diet quality. Partial eta squared was calculated to examine effect sizes. Pearson correlations were used to assess relationships between variables. SPSS (Statistical Package for the Social Sciences) version 25 (IBM Corp., Armonk, NY, USA) was utilized for all statistical procedures, and findings were considered statistically significant at a *p*-value < 0.05.

## 3. Results

### 3.1. Participants

A total of 146 individuals (including 44 vegetarians) completed the online screening questionnaire. Seventeen of the vegetarians met the eligibility criteria and were matched to an omnivore respondent. Thirty-three individuals (17 vegetarians: 3 men/14 women; 16 omnivores: 3 men/13 women) agreed to meet with investigators and were enrolled in the study. Participants were non-smokers, healthy by self-report, not taking prescription medications, and exercised <30 min daily. The median duration of adherence to the vegetarian diet was 27 months. Nearly 79% of study participants were Caucasian, with Hispanics and African Americans representing approximately 15% and 6% of study participants, respectively. Ethnicity did not differ by plant-based diet grouping or by diet quality grouping.

### 3.2. Evaluation by Diet Group

As intended, age, body weight, BMI, waist circumference, and physical activity level did not differ significantly between diet groups (*p* = 0.551, multivariate test; effect size: 0.131). Additionally, plasma folate (a biomarker related to diet quality and to fruit and vegetable intake was 31.7 ± 10.6 and 34.8 ± 13.9 nmol/L for omnivores and vegetarians, respectively; *p* = 0.478), and diet quality scores (37.7 ± 3.1 and 37.8 ± 2.8 for omnivores and vegetarians, respectively; *p* = 0.895) did not differ significantly between diet groups. Blood biomarkers, including plasma fasting insulin and glucose, HOMA-IR, HDL, and LDL cholesterol, total cholesterol, triglycerides (TG), and the TG/HDL ratio, did not differ between groups (*p* = 0.349, multivariate test; effect size: 0.283) (Table 1).

### 3.3. Evaluation by Diet Quality

To examine biomarkers based on diet quality categorization rather than diet adherence, the study sample was reclassified using the median REAP-S score, 37, as the cut-off. Mean diet quality scores were 35.3 ± 2.0 (range: 29–37) and 39.8 ± 1.5 (range: 38–43) for the low (*n* = 15; 8 vegetarians, 4 men) and high (*n* = 18; 9 vegetarians, 2 men) diet quality groups, respectively (*p* < 0.001). Similarly, plasma folate differed significantly following the regrouping: 27.7 ± 10.6 and 38.0 ± 11.9 nmol/L for the low and high diet quality groups, respectively (*p* = 0.014). Age, body weight, BMI, waist circumference, and physical activity level did not differ significantly by diet quality (*p* = 0.520, multivariate test controlling for diet group; effect size: 0.142).

**Figure 1:**
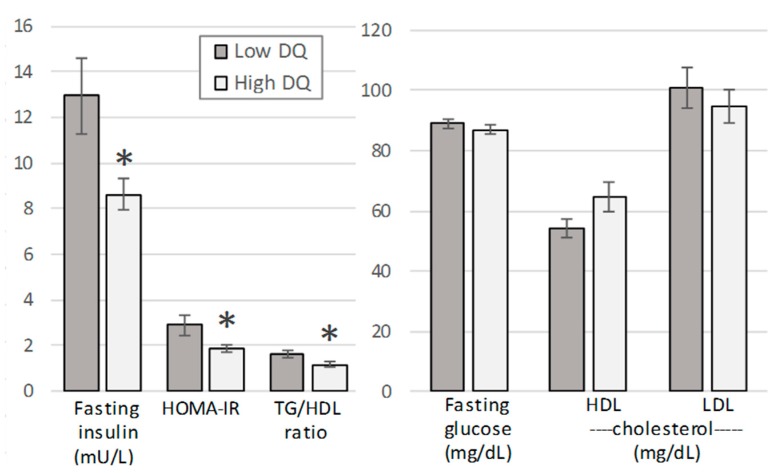
Selected blood biomarkers for diet quality (DQ) groups (low DQ, Rapid Eating and Activity Assessment for Participants Short Version (REAP-S) scores < 37; high DQ, REAP-S scores ≥ 37). Means (±SE) differed significantly by diet quality group (*p* = 0.042, multivariate test; asterisks indicate the significant differences between low and high diet quality groups following post-hoc analysis).

**Figure 2:**
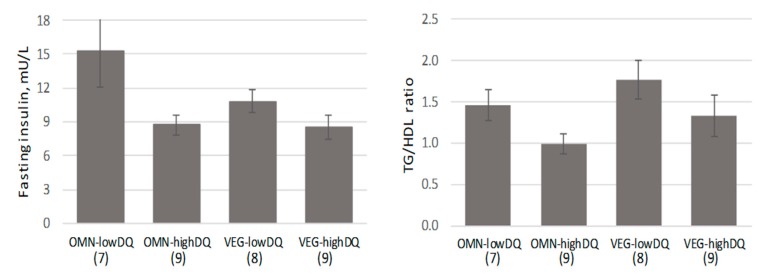
Fasting insulin and triglyceride (TG)/HDL ratio for diet group by diet quality (mean ± SE; *n* in parentheses). Biomarkers differed by diet quality grouping only; there was not a significant interaction for diet group × diet quality group (2-way analysis of variance).

Blood biomarkers differed significantly between the dietary quality groups (*p* = 0.042, multivariate test controlling for diet group; effect size: 0.463). The mean fasting insulin, HOMA-IR, and TG/HDL ratio differed significantly between the diet quality groups, and the high-quality diet values reached only 62–75% of the low-quality diet values (Figure 1). All significant differences noted between diet quality groups were retained when gender was controlled. Figure 2 displays the fasting insulin and the TG/HDL ratio by four groups: Omnivore/low diet quality, omnivore/high diet quality, vegetarian/low diet quality, and vegetarian/high diet quality (*p* > 0.05, two-way analysis of variance for diet group × diet quality).

A subset of six items from the REAP-S questionnaire, items commonly distinguishing healthy vs. unhealthy diets (whole grains, fruits, vegetables, sweets/desserts, sugar-sweetened beverages, and cooking from scratch), were examined for trends by diet or diet quality classification. There were no differences in scores for these items between omnivores and vegetarians. However, when comparing low vs. high diet quality groups, intakes of whole grains, sweets/desserts, and sugar-sweetened beverages as well as the frequency of cooking from scratch, were scored significantly more favorably by the high diet quality group, but the frequency of fruit and vegetable intakes were similar between groups irrespective of the diet or diet quality classification.

## 4. Discussion

These data suggest that several common health biomarkers are more closely aligned with diet quality attributes than with plant-based diet categorization. The REAP-S questionnaire, utilized herein to score diet quality, rated high-quality diets as those rich in vegetables, whole grain/high fiber starches, and low-fat dairy, moderate in lean meats and fish, and largely devoid of processed meats, fried foods, savory snacks, and sweets including sugared drinks. Additionally, foods away from home, highly processed foods, and skipping breakfast are minimized in high-quality diets. These attributes align with the federal nutrition policy and the views of experts [8,9,20]; however, Americans are adopting popular diets focused on a diet premise (e.g., ‘vegan’, ‘paleo’, or ‘low-carb’) and not on diet quality concepts. Thus, messaging focused on high-quality diet attributes may lead to healthier outcomes than the simple promotion of plant-based diets. The data herein suggest that messaging regarding whole grains, sweets, sugar-sweetened beverages, and cooking from scratch might be particularly useful for addressing issues of diet quality.

A recent online nutrition survey conducted by the USDA Human Nutrition Research Center on Aging at Tufts University (Boston, MA) sought respondents who identified as following a popular diet. Of the 9536 individuals who completed the survey, 25% claimed to follow a ‘whole food, plant-based diet’, which ranked first for popular diets [21]. ‘Vegan’ and ‘paleo’ diets ranked second and third in popularity, chosen by 18% and 14% of respondents, respectively, and ‘vegetarian’ was chosen by 9% of the respondents (fifth place). Hence, over 50% of this population reported following plant-based diets. The popularity of plant-based diets may reflect the common belief that these diets are healthy in comparison to meat-based diets [22,23]. When queried for reasons to adopt a ‘more vegetarian diet’ in the future, a predominately omnivore adult population (*n* = 2436) selected ‘my health’ as the most likely reason followed by ‘to discover new tastes’ and ‘to reduce weight’ [24].

Theoretically, plant-based diets are closely aligned with the reference ‘healthy diet’ and with diet quality [25]; yet, much research is emerging suggesting that, in actuality, a portion of individuals following plant-based diets actually have unhealthy eating patterns. In a sub-analysis of the Nurses’ Health Study 2, participants following plant-based diets were fairly evenly partitioned into ‘healthful’ and ‘unhealthful’ dietary patterns based on the predominant plant foods in their diets (whole grains, fruits, vegetables, nuts, legumes, vegetable oil versus fruit juices, refined grains, potatoes, sugar-sweetened beverages, sweets/desserts) [26]. Furthermore, this study demonstrated that favorable profiles for adiposity-associated biomarkers (e.g., insulin, leptin, adiponectin, and CRP) were linked only to ‘healthful’ plant-based diet scores, suggesting that metabolic processes are influenced by plant-based diet categorization [26]. In the present sample, the most favorable profiles for the biomarkers fasting insulin and the TG/HDL ratio were noted for the high diet quality subsets of omnivores and vegetarians (Figure 2). It is interesting to note that the least favorable fasting insulin profile was displayed by omnivores consuming low-quality diets but that the least favorable TG/HDL ratio was displayed by vegetarians consuming low-quality diets. Several trials have cautioned that vegetarian diets may be associated with elevated triglycerides and reduced HDL necessitating particular attention to dietary levels of fructose and other simple carbohydrates [27,28].

In other large population surveys, risks for cardiovascular disease, type 2 diabetes, and all-cause mortality were reduced for the ‘healthy’ plant-based diet group compared to the ‘unhealthy’ plant-based diet group, which supports the contention that not all plant-based diets are equal [10,11,29]. Considering these findings, and that much of the published research on plant-based or vegetarian diets and health outcomes did not account for diet quality [30,31], it is possible that the purported benefits of ‘healthful’ plant-based diets may be moderated and underreported.

This evaluation of diet quality is limited by a small sample size; effect sizes were presented to aid in data interpretation. As a cross-sectional trial, this study can only highlight relationships between variables and cannot demonstrate causality, and the dietary data were self-reported and limited in scope. However, a biomarker for diet quality, plasma folate, did correlate moderately with diet quality scores (*r* = 0.47; *p* = 0.006) suggesting concurrent validity. Although the REAP-S questionnaire has been validated [15,16,17], it is not in widespread use, and scoring norms are unavailable. Herein, the average diet quality score overall, 37, fell at 73% in the range of scores (15–45). In comparison, HEI scores ranging from 51–80% of the total score reflect diets that ‘needs improvement’, and the average American HEI score was 59% in 2013–2014 [32].

## 5. Conclusions

In this investigation, health biomarkers did not differ between vegetarians and omnivores matched for gender, age, and adiposity. However, when participants were regrouped by low versus high diet quality, health biomarkers differed significantly between groups for fasting insulin, HOMA-IR, TG/HDL ratio, and blood folate, with more favorable levels in the group with high diet quality scores. These data suggest that diet quality attributes are more closely aligned with health biomarkers than plant-based diet categorization. It is encouraging that plant-based diets appear to be the most popular diet trend in the U.S.; yet, about one-half of those adopting plant-based diets are not consuming ‘healthful’ diets and may not benefit from reduced chronic disease risk. Healthcare practitioners should emphasize diet quality in their messages to their clients and discuss the importance of eating whole, minimally processed foods with less added fat and sugars. The REAP-S questionnaire is a simple tool that could help practitioners achieve this goal. Additionally, since plant-based diets support the environment and food system sustainability [33,34,35], these attributes should be included in messaging.

## Figures and Tables

**Table 1:** Participant characteristics ^1^.

Characteristic	Total Sample (*n* = 33; 6 M, 27 F)	Vegetarians (*n* = 17; 3 M, 14 F)	Omnivores (*n* = 16; 3 M, 13 F)
Age, year	28.2 ± 8.9	27.1 ± 8.9	29.4 ± 9.1
Body weight, kg	63.4 ± 8.8	62.0 ± 8.2	64.8 ± 9.4
Body mass index, kg/m^2^	22.5 ± 2.7	21.9 ± 2.5	23.2 ± 2.8
Waist circumference, cm	77.0 ± 11.9	76.4 ± 15.1	77.6 ± 7.7
METS, kcal/kg·wk	52.2 ± 27.3	53.9 ± 24.6	50.5 ± 30.7
Diet Quality, score	37.8 ± 2.9	37.8 ± 2.8	37.7 ± 3.1
Plasma folate, nmol/L	33.3 ± 12.3	34.8 ± 13.9	31.7 ± 10.6
Fasting glucose, mg/dL	87.8 ± 5.5	85.6 ± 4.7	90.2 ± 5.5
Fasting insulin, mU/L	10.6 ± 5.2	9.6 ± 3.2	11.6 ± 6.7
HOMA-IR, score	2.3 ± 1.3	2.0 ± 0.7	2.6 ± 1.7
Triglycerides, mg/dL	75.2 ± 25.3	77.9 ± 26.4	72.4 ± 24.5
Total cholesterol, mg/dL	165.6 ± 31.7	163.1 ± 29.8	168.3 ± 34.4
HDL cholesterol, mg/dL	60.0 ± 17.8	56.2 ± 16.2	64.0 ± 18.9
LDL cholesterol, mg/dL	97.5 ± 23.9	100.6 ± 25.1	94.2 ± 23.0
TG/HDL ratio	1.37 ± 0.63	1.54 ± 0.73	1.19 ± 0.47

^1^ Data are mean ± SD; characteristics did not differ significantly between diet groups (*p* > 0.05). M, male; F, female; METS, metabolic equivalent of task.
